# Supplementary material for: Self-amplified Amazon forest loss due to vegetation-atmosphere feedbacks
Source: Nat Commun. 2017 Mar 13;8:14681. doi: 10.1038/ncomms14681 (PMC5355804; doi:10.1038/ncomms14681)
Supplement: Supplementary Information — Supplementary Figures and Supplementary Tables [file ncomms14681-s1.pdf]

Data	Δ E (mm yr^{-1})	Δ P (mm yr^{-1})	Δ E (%)	ΔP (%)
All	-108 (-803)	-32 (-274)	-9 (-52)	-2 (-13)
Reanalyses	-34 (-1068)	-11 (-371)	-3 (62)	-1 (-17)
Diagnostic	-153 (-941)	-52 (-326)	-11 (-56)	-3 (-15)
LSM	-118 (-929)	-31 (-312)	-11 (-64)	-2 (-15)

Supplementary Table 1. Effect of Amazon deforestation on rainfall and evapotranspiration. Changes in annual evapotranspiration over the deforested area (ΔE) and annual rainfall over the Amazon basin (ΔP) as simulated by the coupling of moisture recycling networks and the evapotranspiration model (step 1 of the cascade model). Results are shown for different categories of LandFlux-Eval evapotranspiration datasets: all categories, reanalyses, satellite and/or ground-based observations (diagnostic) and land-surface model outputs (LSM). Upper uncertainty bound associated with the 95% confidence interval of evapotranspiration change estimates is shown in brackets.

	Current	End of 21st century	LGM	-6mm d^{-1} in June–Nov.
$\phi = 0.4$	0 (0)	1 (1.4)	13.2 (18.5)	19.1 (38.0)
$\phi = 0.5$	0 (0)	3.5 (14.3)	9.8 (21.9)	14.6 (30.3)
$\phi = 0.6$	1.2 (2.8)	7.0 (13.2)	11.8 (22.6)	10.8 (25.4)

Supplementary Table 2. Self-amplified forest loss with dry-season oceanic moisture inflow reduction. Additional forest loss due to self-amplifying effects (in % of the total Amazon basin) for different resilience thresholds (ϕ) and for different oceanic moisture inflow scenarios: current conditions, end of 21st century scenario, LGM scenario and breakdown of dry-season oceanic moisture inflow (-6 mm d^{-1} in June–November). Upper uncertainty bound associated with the 95% confidence interval of evapotranspiration change estimates is shown in brackets.

**Oceanic moisture inflow scenario**			
Initial vegetation	Figure/Table	Method/model	Spatial res.
**Current oceanic moisture inflow**			
Current vegetation	Fig. 2a	Frequency distribution	0.25°
	Fig. 2b	Logistic regression	0.25°
	Fig. 3c	WAM-2layers	1.5°
	Fig. 3a	Logistic regression	1.5°
	Suppl. Figs. 12 and 13	P↔veg	1.5°
	Suppl. Fig. 10	Budyko curve	1°
	Suppl. Fig. 1	Potential landscape	0.25°
100% deforestation	Fig. 3b, Suppl. Table 1	veg→P	1.5°
	Suppl. Fig. 3	GLM	1°
	Suppl. Fig. 2	Evap. model based on Gerrits'model	1°
**Gradual dry-season oceanic moisture inflow decrease**			
Current vegetation	Figs. 4a, Suppl. Fig. 6	P→veg and P↔veg	1.5°
	Figs. 4b, Suppl. Fig. 5a	P→veg	1.5°
	Suppl. Fig. 5b	veg→P with GLM	1.5°
100% deforestation	Figs. 4c, Suppl. Fig. 5c	veg→P	1.5°
	Suppl. Fig. 5d	veg→P with GLM	1.5°
**End of 21st century scenario**			
Current vegetation	Fig. 5a, Suppl. Figs. 7a, 8a	P↔veg	1.5°
	Figs.5b-c, Suppl. Figs. 7b-c, 8b-c	P→veg and P↔veg	1.5°
**LGM scenario**			
Current vegetation	Fig. 5d, Suppl. Figs. 7d, 8d	P↔veg	1.5°
	Figs. 5e-f, Suppl. Figs. 7e-f, 8e-f	P→veg and P↔veg	1.5°
**Breakdown of dry-season oceanic moisture inflow (-6mm d^{-1} in June–November)**			
Current vegetation	Fig. 6, Suppl. Fig. 9	P↔veg	1.5°

Supplementary Table 3. Overview of model settings, scenarios, initial vegetation conditions and spatial resolution for the different figures and tables. Unless stated otherwise, the cascade model runs with the evapotranspiration model based on Gerrits' model. Abbreviations: res.: resolution; evap.: evapotranspiration; Suppl: Supplementary.

High TC	all		diagnostic		reanalyses		LSM	
p3	-1.0993	(0.0211)	-1.2107	(0.0258)	-1.0375	(0.0362)	-0.8617	(0.0245)
p4	2.7081	(0.0175)	3.1618	(0.0192)	2.2627	(0.0211)	2.2466	(0.2267)
p1	2.1145	(0.1189)	2.9326	(0.1833)	2.7412	(0.1087)	0.0141	(0.0106)
p2	2.1317	(0.0996)	3.2089	(0.1863)	5.4777	(0.2238)	0.0113	(0.0057)
MSE	0.1581		0.2228		0.4755		0.2284	
**Inter. TC**	**all**		**diagnostic**		**reanalyses**		**LSM**	
p3	-0.3294	(0.0092)	-0.3518	(0.0099)	-0.2480	(0.0136)	-0.3592	(0.0099)
p4	1.7672	(0.0108)	2.0928	(0.0118)	1.6874	(0.0150)	1.4587	(0.0128)
p1	1.8493	(0.0368)	1.8277	(0.0415)	2.2101	(0.0555)	1.4670	(0.0390)
p2	4.5885	(0.1096)	4.4088	(0.1209)	6.4417	(0.2214)	2.9756	(0.0830)
MSE	0.2925		0.3309		0.6471		0.3436	
**Treeless**	**all**		**diagnostic**		**reanalyses**		**LSM**	
p3	-0.2911	(0.0091)	-0.2811	(0.0101)	-0.3243	(0.0119)	-0.2769	(0.0104)
p4	1.5490	(0.0226)	1.8942	(0.0243)	1.5981	(0.0303)	1.0203	(0.0273)
p1	1.5971	(0.0635)	1.8897	(0.0888)	1.3782	(0.0697)	1.3174	(0.0606)
p2	3.8513	(0.1798)	4.2647	(0.2461)	3.4469	(0.1986)	3.1712	(0.1591)
MSE	0.2973		0.3605		0.5035		0.3896	

Supplementary Table 4. **Parameter estimates of the evapotranspiration model for different input datasets**. Best estimates and standard errors for the 95% confidence bound (in brackets) are shown for different categories of LandFLUX-EVAL evapotranspiration datasets: satellite and ground-based observations (diagnostic), land-surface model outputs (LSM), reanalyses and all categories together. Results are shown for the different tree-cover (TC) states: high, intermediate (int.) and treeless. The goodness of fit is shown using the mean square errors (MSE). Note that errors of the parameters are very low (less than 5% in most of the cases), which means that the model fits well to the data, regardless of the chosen datasets. Certain parameter estimates vary depending on the choice of the input data (for example, estimates of p_2 for the intermediate tree-cover state for reanalyses data differ from estimates using land-surface model outputs). However, this variability does not affect our results regarding the effects of environmental perturbations on evapotranspiration as described in the last subsection of the Results section.

	CRU	TRMM
β_0	2.0128	-1.9227
β_1	0.0003	0.0015
β_2	0.0074	0.0036

Supplementary Table 5. **Parameters of the logistic regression predicting the probability of forest cover as a function of rainfall regime for different precipitation input data.** All parameters are statistically significant (p value < 0.05).

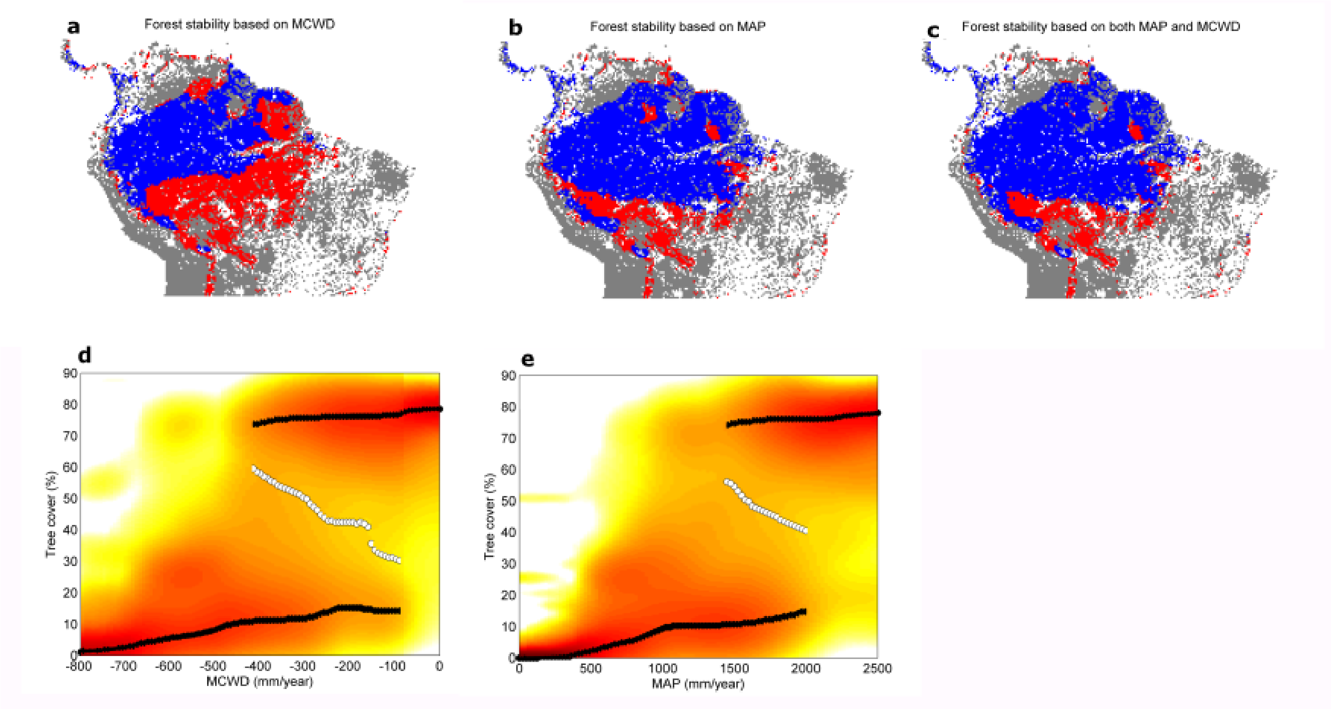

Supplementary Figure 1. Alternative tree-cover states in relation to rainfall regimes. Range where the forest is bistable (red) and stable (blue) computed from the data using (**a**), the maximum climatological water deficit (MCWD), (**b**), the mean annual precipitation (MAP) and (**c**), the combination of MAP and MCWD using monthly rainfall data (TRMM 3B42 for the period 2000-2012). Potential landscape of tree cover in relation to (**d**), MCWD and (**e**), MAP. Solid dots correspond to stable states and open dots to unstable states. Reddish colours indicates regions of high point density at a given value of MAP or MCWD.

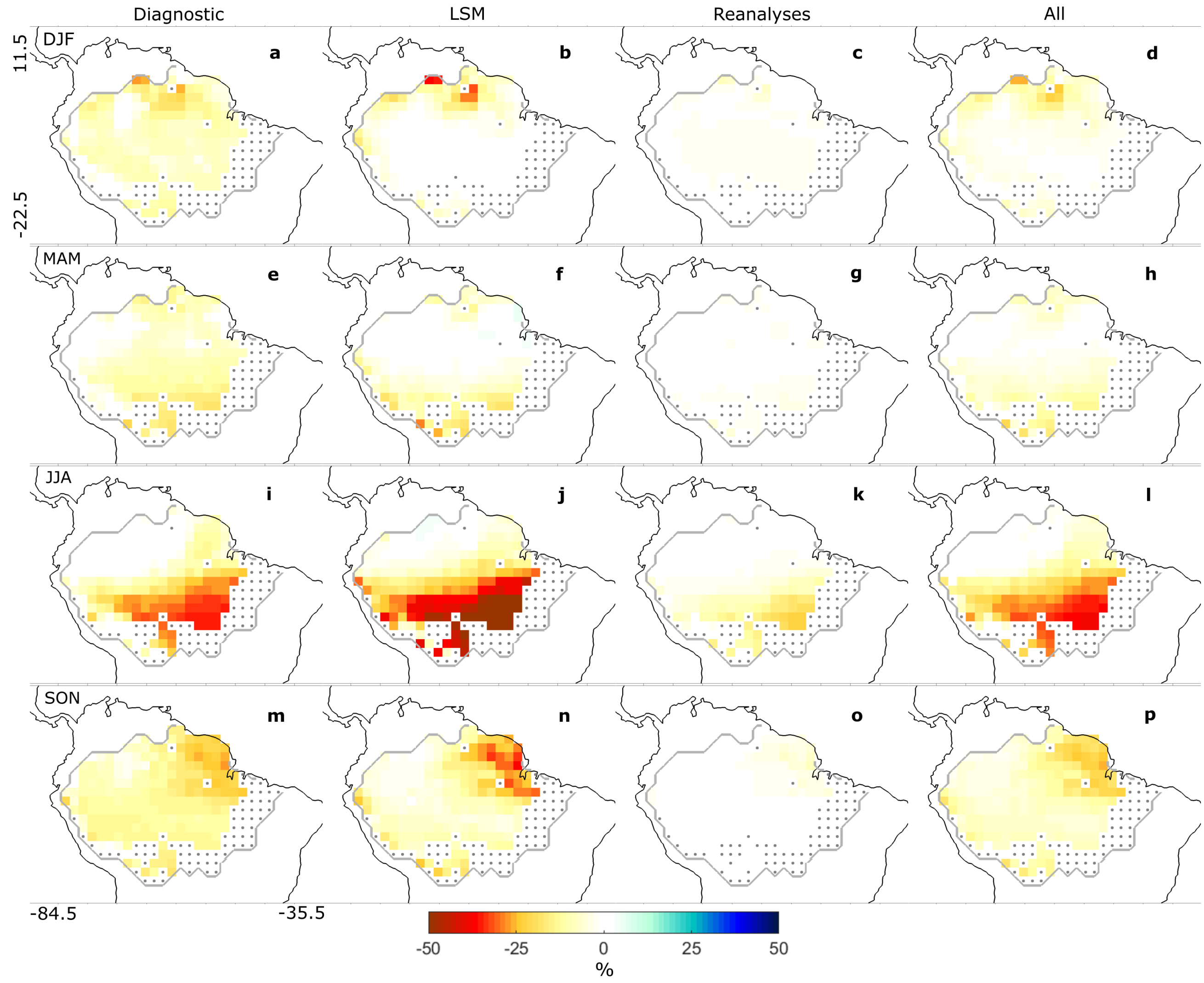

Supplementary Figure 2. Effect of complete Amazon forest loss on seasonal evapotranspiration for different input datasets using the simple evapotranspiration model. Changes in evapotranspiration after complete Amazon deforestation for **(a,b,c,d)** December, January and February (DJF), **(e,f,g,h)** March, April and May (MAM), **(i,j,k,l)** June, July and August (JJA) and **(m,n,o,p)** September, October and November (SON). Results are shown for different categories of LandFlux-EVAL evapotranspiration datasets: **(a,e,i,m)** satellite and ground-based observations (diagnostic), **(b,f,j,n)** land-surface model outputs (LSM), **(c,g,k,o)** reanalyses and **(d,h,i,p)** all categories.

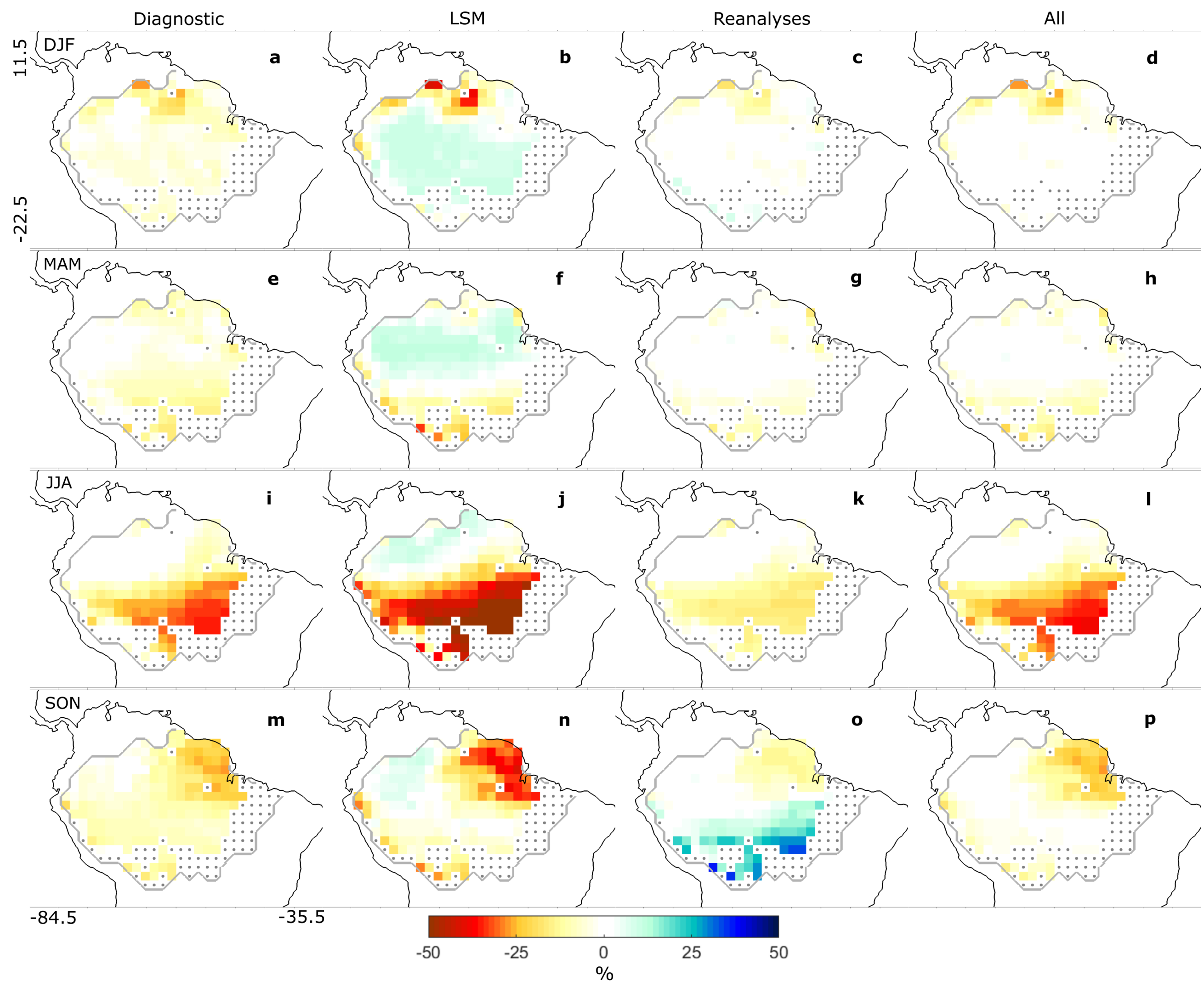

Supplementary Figure 3. Effect of complete Amazon forest loss on seasonal evapotranspiration for different input datasets using the generalized linear model. Changes in evapotranspiration after complete Amazon deforestation for **(a,b,c,d)** December, January and February (DJF), **(e,f,g,h)** March, April and May (MAM), **(i,j,k,l)** June, July and August (JJA) and (m,n,o,p) September, October and November (SON). Results are shown for different categories of LandFLUX-EVAL evapotranspiration datasets: **(a,e,i,m)** satellite and ground-based observations (diagnostic), **(b,f,j,n)** land-surface model outputs (LSM), **(c,g,k,o)** reanalyses and **(d,h,l,p)** all categories.

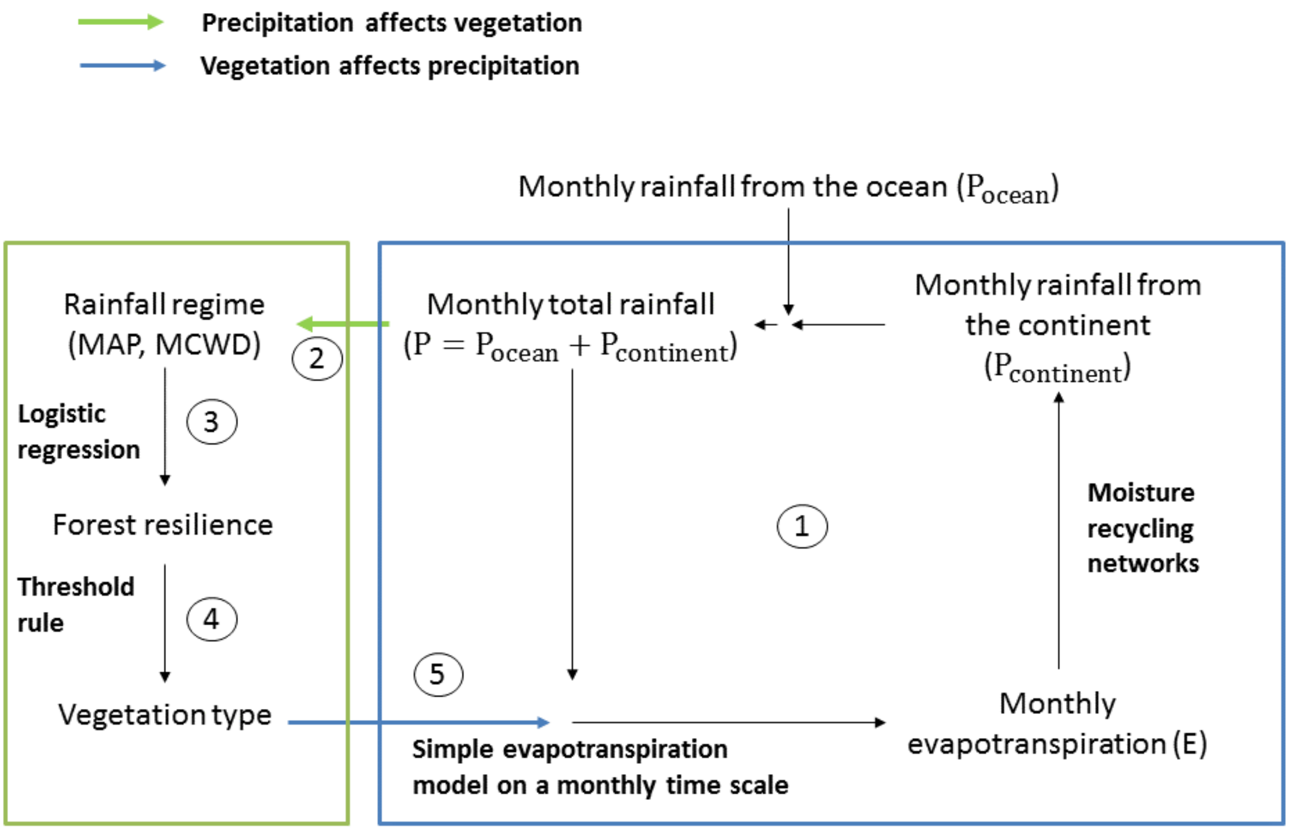

Supplementary Figure 4. Cascade model scheme. A simulation run comprises the following steps: (1) Moisture of oceanic origin propagates through the network. (2) Rainfall regime characterized by the mean annual precipitation (MAP) and Maximum Cumulative Water Deficit (MCWD) is calculated on a grid-cell basis. (3) Forest resilience is calculated for each grid cell using the output of a logistic regression on remotely sensed data. (4) A critical transition occurs in each grid cell for which the forest resilience crosses the individual threshold. (5) Local evapotranspiration is updated in grid cells where shifts occur. If mode "veg↔P" (see Methods) and if there are shifts in the vegetation, go to step 1 and continue the iterations until equilibrium is reached. If mode is "P→veg", stop here. If mode is "veg→P", start with step 5, continue with step 1 and stop here (steps 2-4 are omitted). Steps 1 and 5 occur on a monthly time scale, whereas steps 2, 3 and 4 occur on an annual time scale.

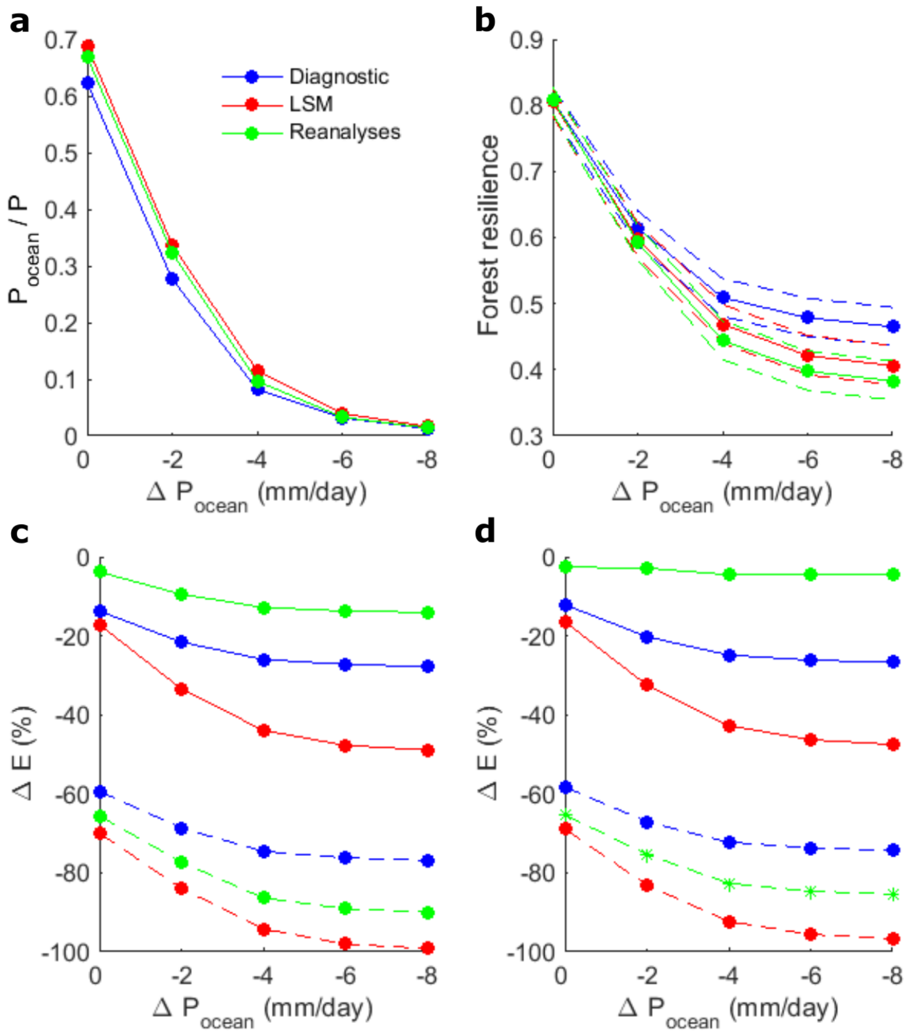

Supplementary Figure 5. Sensitivity of the results to the choice of evapotranspiration input data and model. Various variables as a function of monthly oceanic moisture inflow reduction during the extended dry season (ΔP_{ocean}). Results are shown for different evapotranspiration input datasets (diagnostic, reanalyses and land-surface model, see Methods). (**a**) Fraction of rainfall that last evaporated from the ocean. (**b**) Resilience of Amazon forest. **c-d** Changes in evapotranspiration after complete deforestation based on (**c**) our simple evapotranspiration model and (**d**) the generalized linear model. Dashed lines show the 95% confidence intervals.

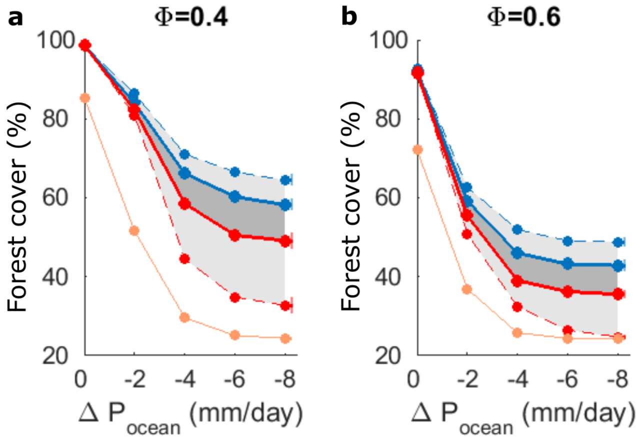

Supplementary Figure 6. Sensitivity of the results to mean resilience thresholds. Fraction of the Amazon basin covered by forest (median of all realizations as lines, 95% bounds shown by coloured bars on the right side of the plot). The difference between forest cover using the one-way coupling "P→veg" (blue line) and the fully coupled system "P↔veg" (red line) quantifies the additional forest loss due to self-amplifying effects (gray area). Results obtained considering the 95% confidence bound in estimated evapotranspiration change after forest loss are shown (dashed lines). For comparison, the maximum possible forest loss for a complete failure of moisture recycling is also shown (orange line). Results are shown for (**a**) $\Phi = 0.4$ and (**b**) $\Phi = 0.6$.

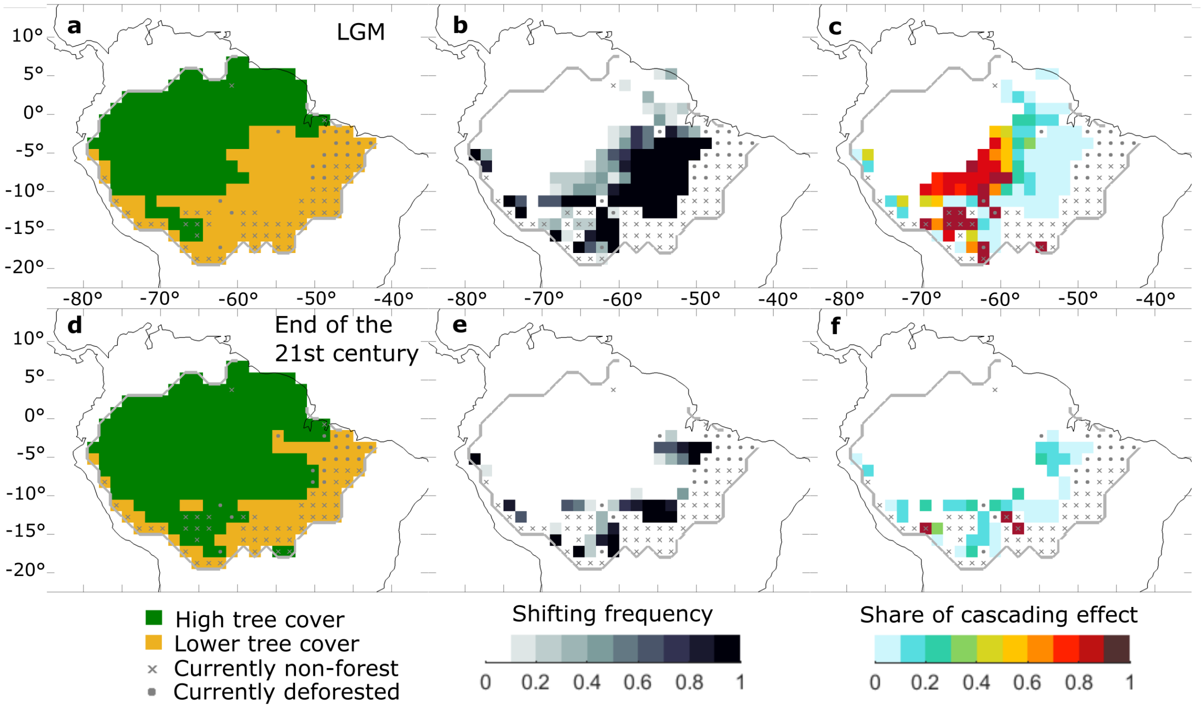

Supplementary Figure 7. Self-amplified forest loss for the Last Glacial Maximum (LGM) and the end of the 21st century for mean resilience threshold $\Phi = 0.4$. (a,d) Most frequent vegetation cover for 1000 realizations of the cascade model. **(b,e)** Shifting frequency of Amazon forest. **(c,f)** Share of cascading effects in causing forest shifts (see Methods). Results are shown **(a,b,c)** for the LGM scenario and **(d,e,f)** for the "end of 21st century" scenario (see Methods).

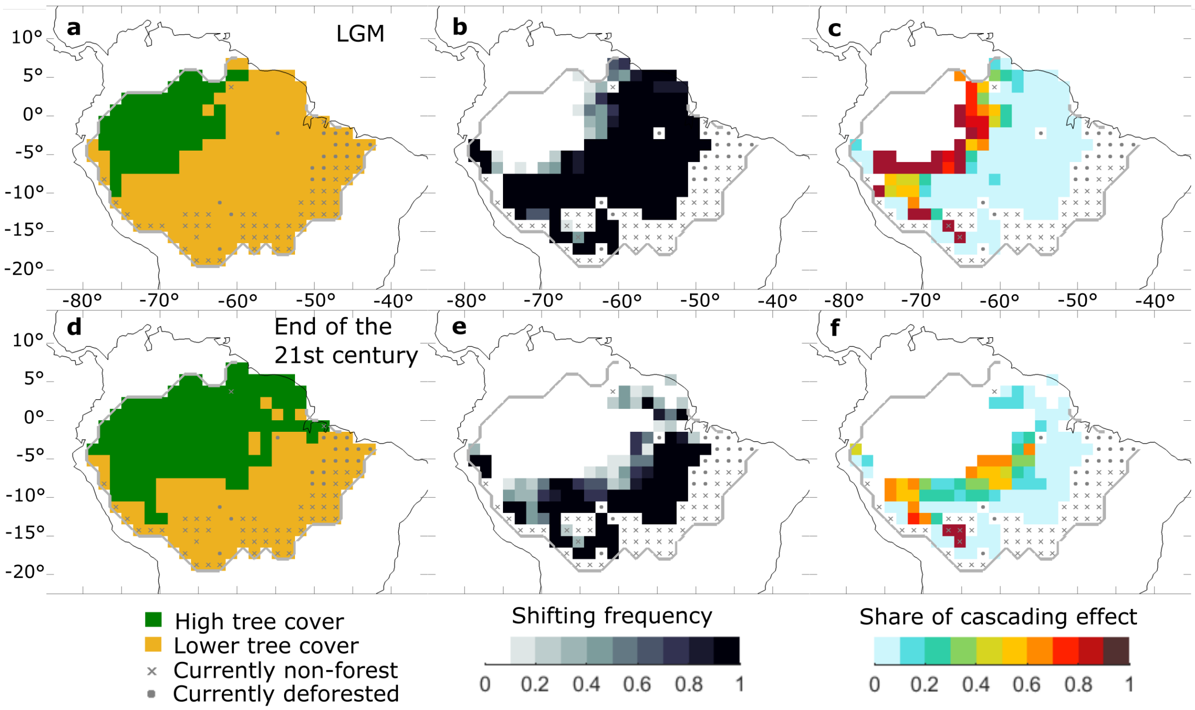

Supplementary Figure 8. Self-amplified forest loss for the Last Glacial Maximum (LGM) and the end of the 21st century for mean resilience threshold $\Phi = 0.6$. (a,d) Most frequent vegetation cover for 1000 realizations of the cascade model. **(b,e)** Shifting frequency of Amazon forest. **(c,f)** Share of cascading effects in causing forest shifts (see Methods). Results are shown **(a,b,c)** for the LGM scenario and **(d,e,f)** for the "end of 21st century" scenario (see Methods).

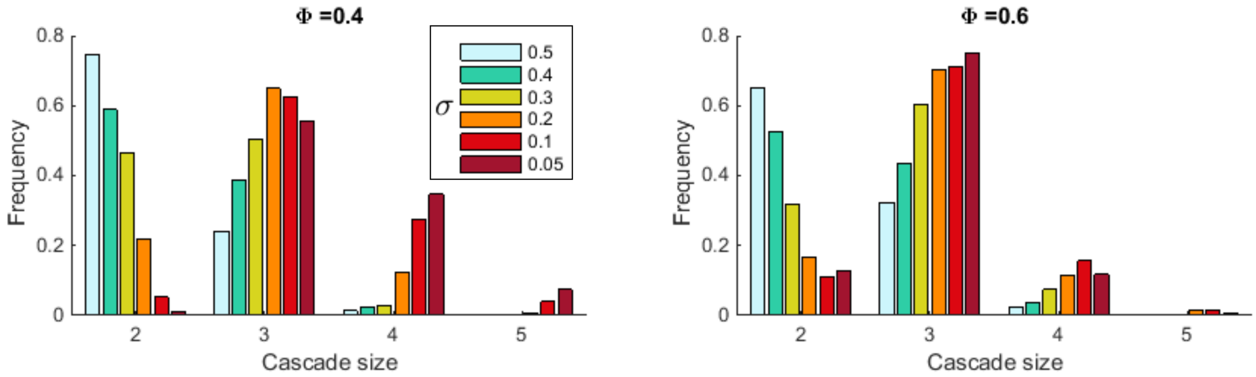

Supplementary Figure 9. Effect of heterogeneity on the stability of the vegetation-rainfall system for different values of Φ. Frequency distribution of cascade sizes with increasing heterogeneity of the forest resilience thresholds based on 1000 realizations of the cascade model. Note that with increasing heterogeneity (σ), the frequency of high-order cascades (cascade size = 4) decreases and the frequency of low-order cascades (cascade size = 2) increases.

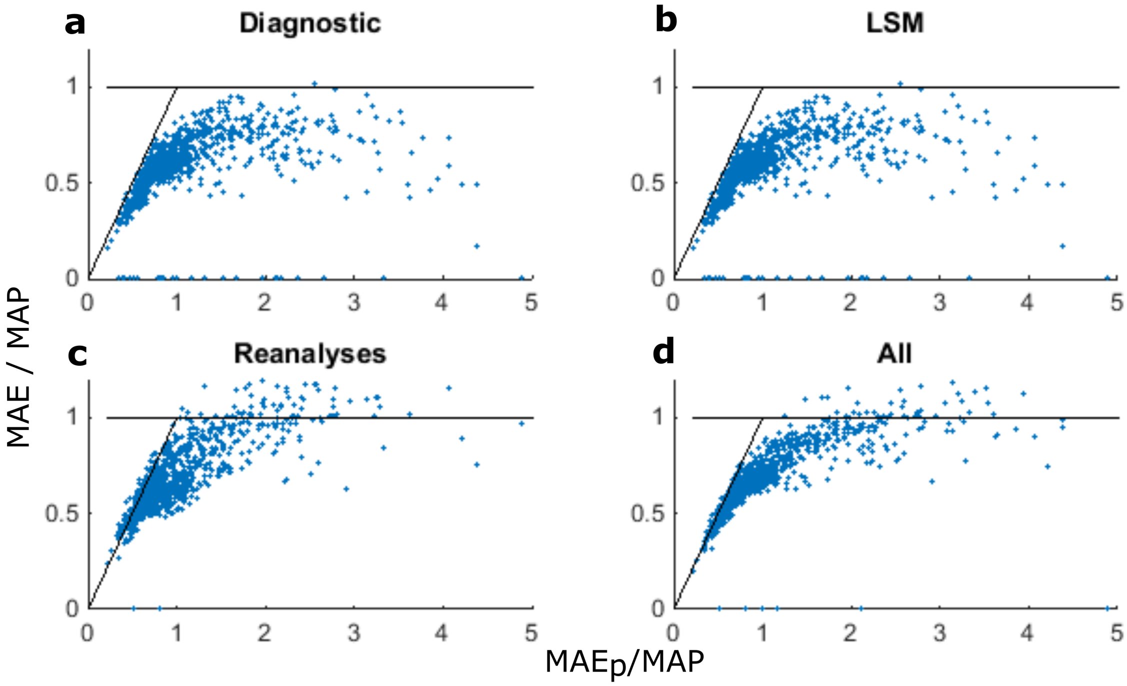

Supplementary Figure 10. Budyko curves showing agreements between hydro-climate datasets. Abbreviations: mean annual evapotranspiration (MAE), mean annual precipitation (MAP), mean annual potential evapotranspiration (MAE_p). The 1:1 line shows the limitation by available energy ($MAE_p < MAE$), and the horizontal line shows the limitation by available water ($MAE_p > MAP$). Results are shown for different categories of LandFLUX-EVAL evapotranspiration datasets: **(a)** satellite and/or measurements (diagnostic), **(b)** land-surface model outputs (LSM), **(c)** output from atmospheric reanalyses and **(d)** all categories.

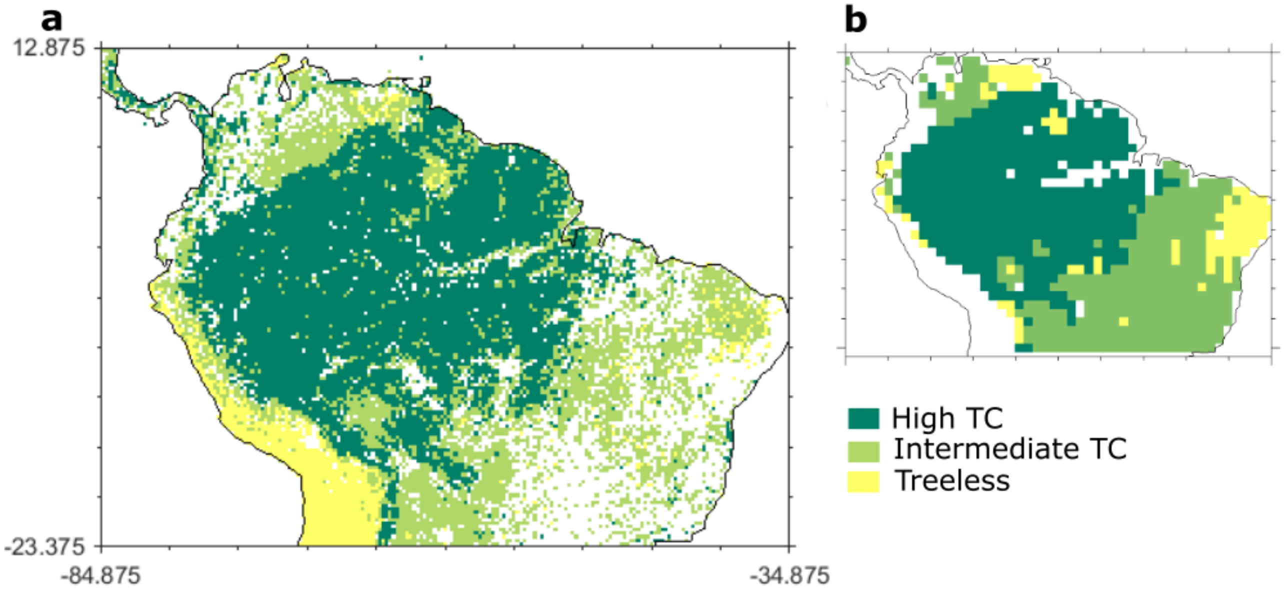

Supplementary Figure 11. Classification of tree-cover states at different spatial resolutions. Classification of tree-cover (TC) states **(a)** at 0.25° resolution, used for calculating the forest resilience, and **(b)** at 1° resolution, used for calibrating the evapotranspiration model. Note that the grid cells excluded from the analysis (in white) are not the same (see Methods).

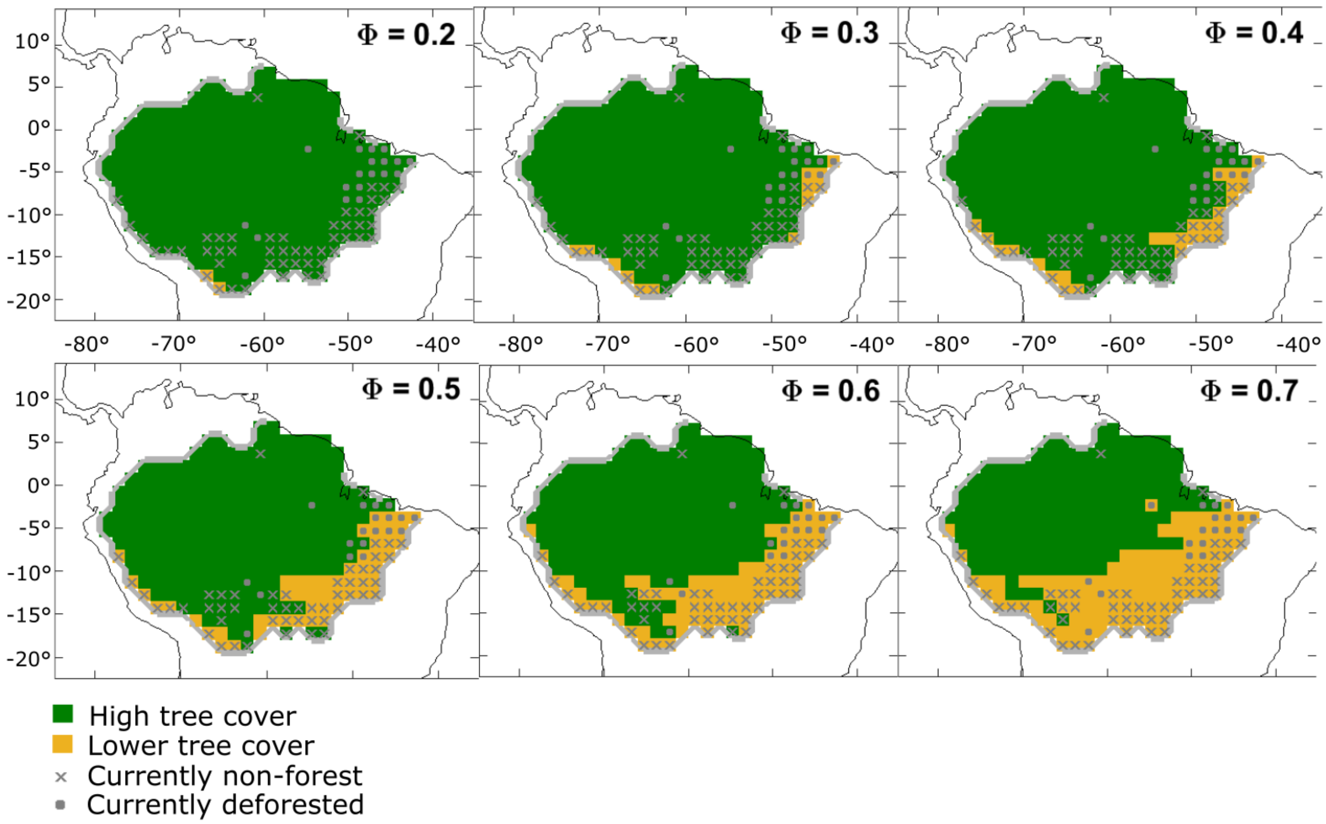

Supplementary Figure 12. **Vegetation cover under current rainfall regime (1989-2005) for different resilience thresholds' mean values (Φ).**

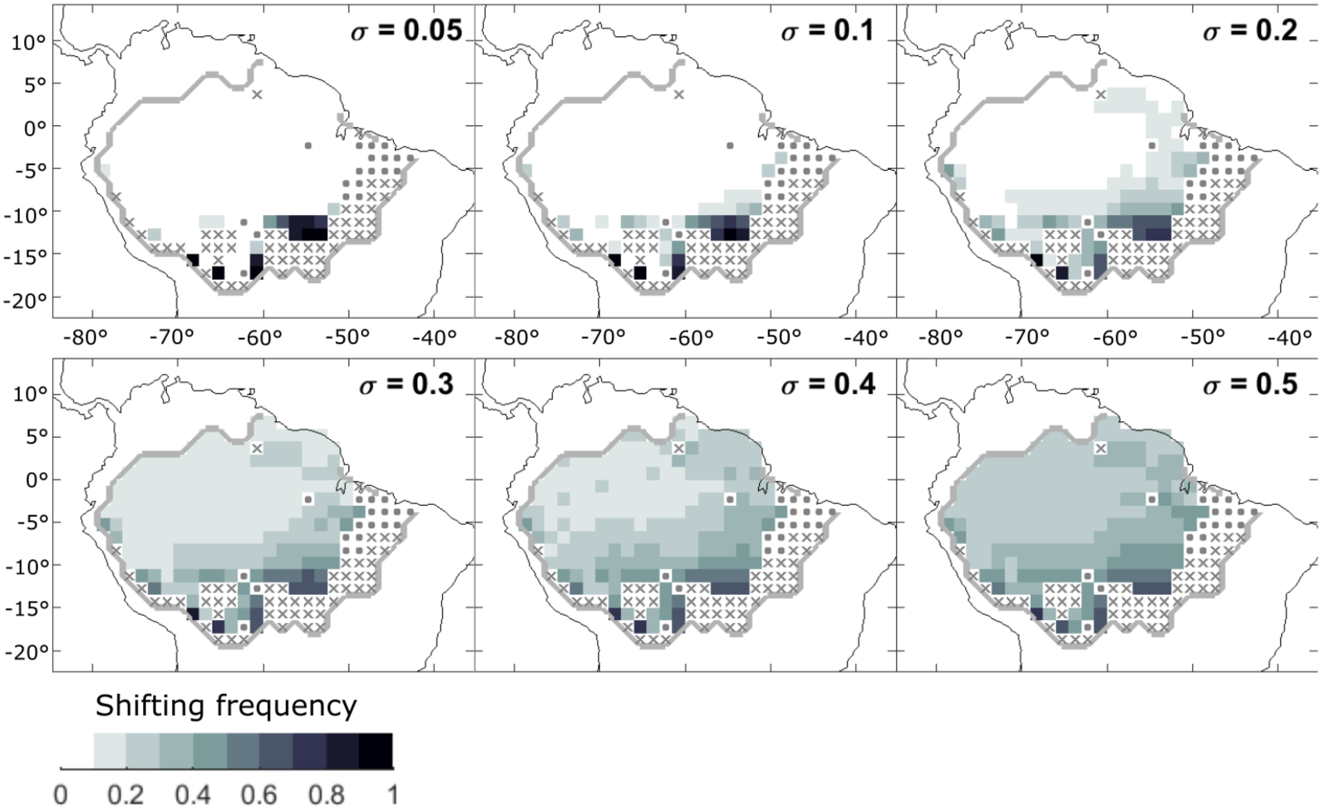

Supplementary Figure 13. **Shifting frequency under current rainfall regime (1989-2005) for different resilience thresholds' deviations (σ).**
